# The RE-AIM framework-based evaluation of the implementation of the Maternal and Child Health Handbook program in Angola: a mixed methods study

**DOI:** 10.1186/s12913-022-08454-9

**Published:** 2022-08-22

**Authors:** Ai Aoki, Keiji Mochida, Michiru Kuramata, Toru Sadamori, Aliza K C Bhandari, Helga Reis Freitas, João Domingos da Cunha, Ketha Rubuz Francisco, Pedro Sapalalo, Lino Tchicondingosse, Olukunmi Omobolanle Balogun, Hirotsugu Aiga, Kenji Takehara

**Affiliations:** 1 Department of Health Policy, National Center for Child Health and Development, 2-10-1 Okura, Setagaya, Tokyo, Japan; 2 TA Networking Corp., 2-7 Nanpeidai, Shibuya, Tokyo, Japan; 3 Department of Global Health, Graduate School of Health Sciences, University of the Ryukyus, 207 Uehara, Nishihara, Nakagami, Okinawa Japan; 4 Samauma Consulting LLC, Chiba 400-5 Nedo, Kashiwa, Japan; 5 St Luke’s International University, 10-1 Akashicho, Chuo, Tokyo, Japan; 6 National Directorate of Public Health, Ministry of Health, Rua 1º Congressodo MPLA Nº67, Luanda, Angola; 7 Domus Custodius (SU) Lda. Tchikos Agency, Via Expressa de Cacuaco, Nova Urbanização II, Cacuaco, Luanda, Angola; 8 School of Tropical Medicine and Global Health, Nagasaki University, 1-12-4, Sakamoto, Nagasaki, Nagasaki Japan; 9 Human Development Department, Japan International Cooperation Agency, 5-25, Nibancho, Tokyo, Chiyoda Japan

**Keywords:** Maternal and child health, Maternal and child health handbook, Home-based record, Developing country, Angola, Implementation, RE-AIM framework, The Consolidated Framework for Implementation Research

## Abstract

**Background:**

The World Health Organization recommends the Maternal and Child Health Handbook (MCH-HB) to promote health service utilization from pregnancy to early childhood. Although many countries have adopted it as a national health policy, there is a paucity of research in MCH-HB’s implementation. Thus, this study aimed to evaluate the MCH-HB’s implementation status based on the RE-AIM framework (Reach, Effectiveness, Adoption, Implementation, Maintenance), and identify facilitators of, and barriers to its implementation in Angola to understand effective implementation strategies.

**Methods:**

A cross-sectional survey was conducted targeting all health facilities which implemented MCH-HB, subsamples of health workers, and officers responsible for the MCH-HB at the municipality health office. Using the 14 indicators based on the RE-AIM framework, health facilities’ overall implementation statuses were assessed. This categorized health facilities into optimal-implementation and suboptimal-implementation groups. To identify barriers to and facilitators of MCH-HB implementation, semi-structured interviews were conducted among health workers and municipality health officers responsible for MCH-HB. The data were analyzed via content analysis.

**Results:**

A total of 88 health facilities and 216 health workers were surveyed to evaluate the implementation status, and 155 interviews were conducted among health workers to assess the barriers to and facilitators of the implementation. The overall implementation target was achieved in 50 health facilities (56.8%). The target was achieved by more health facilities in urban than rural areas (urban 68.4%, rural 53.6%) and by more health facilities of higher facility types (hospital 83.3%, health center 59.3%, health post 52.7%). Through the interview data’s analysis, facilitators of and barriers to MCH-HB were comprehensively demonstrated. MCH-HB’s content advantage was the most widely recognized facilitator and inadequate training for health workers was the most widely recognized barrier.

**Conclusions:**

Strengthening education for health workers, supervision by municipality health officers, and community sensitization were potential implementation strategies. These strategies must be intensified in rural and lower-level health facilities.

**Supplementary Information:**

The online version contains supplementary material available at 10.1186/s12913-022-08454-9.

## Background

Maternal and child health—especially maternal, neonatal, and infant mortality—is among the highest public health priorities in many low- and middle-income countries (LMICs). The United Nations’ Sustainable Development Goals (SDGs) list maternal and child health at the top of SDG 3: Ensure healthy lives and promote well-being for all ages [1]. However, many LMICs have not achieved their targets with respect to maternal and child health yet, especially those in Sub-Saharan Africa [2, 3].

To improve maternal and child health, the promotion of a continuum of care (CoC) from pregnancy and delivery to early childhood is essential along with providing essential lifesaving services [4, 5]. The education of mothers, families, and communities is a key intervention method for the promotion of CoC, especially in LMICs [6].

The Maternal and Child Health Handbook (MCH-HB) is an integrated home-based record (HBR), which records all the key information and data on health service utilization and health conditions of a mother and her child during the course of pregnancy, delivery, and after birth (e.g., maternal care and the child’s growth and immunizations) [7, 8]. The World Health Organization (WHO) along with some studies recommended MCH-HB as one form of HBR to improve health service utilization [9–12]. Furthermore, the MCH-HB functions as a self-learning resource, helps avoid multiple HBRs [13], and supports improvements in CoC [14–16]. Therefore, the MCH-HB has drawn greater attention from health ministries and professional organizations across the globe for being an effective tool for promoting a life course approach to healthcare [8]. It has been introduced in more than 50 countries (e.g., Indonesia, Mongolia, the Philippines, and Sudan) [7, 17].

The MCH-HB program was adopted in Angola under a national health policy to increase CoC, with technical support from the Japan International Cooperation Agency (“Project for Improving Maternal and Child Health Services through the implementation of the Maternal and Child Health Handbook”). The MCH-HB program is a package of MCH-HB distribution, health worker education, and community sensitization that enables its effective utilization. Preceding its nationwide scale-up, a cluster randomized controlled trial (MCH-HB RCT) aimed at estimating the impact of the MCH-HB program on CoC achievements was conducted in a province in Angola, starting in June, 2019 [18].

A better implementation of evidence-based interventions is the key to health promotion in LMICs [19]. Although the MCH-HB is usually delivered together with education for health workers to ensure its appropriate use, there is a dearth of evidence related to the implementation of the MCH-HB program based on implementation science [9, 20]. Thus, this study aimed to evaluate the implementation status of the MCH-HB program and its barriers and facilitators in the intervention group of the MCH-HB RCT to better understand the program’s effective implementation strategies. This study provides useful insights into more effective implementation strategies for the MCH-HB program to provincial health departments in Angola and other countries implementing MCH-HB. To the authors’ knowledge, this is the first study to examine the implementation status of the MCH-HB and the barriers to and facilitators for its implementation using a theoretical framework for implementation research.

## Methods

### Study setting

This study was conducted in Benguela province in Angola, which is a lower-middle-income country in sub-Saharan Africa [21]. According to WHO, approximately 241 maternal deaths occurred per 100,000 live births in 2017 [2]. This was primarily due to preventable diseases and other health problems. In addition, Angola remains one of the African countries with the highest burden of under-five mortality (81 per 1000 live births) and infant mortality rates (54 per 1000 live births), despite a consistent reduction in recent years [3]. Reasons for such a situation can be attributed to factors like lower functioning health systems and shortfalls in the health workforce [22, 23].

Benguela Province is in the southwest of the country, facing the Atlantic Ocean. Benguela has 10 administrative divisions called municipalities, with a population of approximately 2.2 million [24]. Being the third most populous province in Angola, Benguela was deliberately selected as a site for the MCH-HB RCT because data on major health indicators of this province are similar to the national average. Out of 10 municipalities in Benguela, five were randomly allocated to the intervention group of the MCH-HB RCT. The intervention of the MCH-HB RCT was a package of distribution of the MCH-HB to pregnant women at health facilities, training of health workers on the MCH-HB operation, and community sensitization targeting pregnant women on the MCH-HB use. The MCH-HB program was conducted in all health facilities under the jurisdiction of the Ministry of Health that provided maternal, neonatal, and child health services (MNCH services) in the intervention group. Health facilities were categorized into three levels according to the services they provided: health posts (primary healthcare services), health centers (laboratory services and 24-h delivery), and hospitals (specialized services). The MCH-HB RCT began in June 2019, and concluded with an end line survey on October 2020. Women who became pregnant between March and April 2019 and who utilized any MNCH services were enrolled in the study.

### Study design

This was a cross-sectional observational survey which conducted along with the MCH-HB RCT. In this study, health facilities’ overall implementation status was evaluated, and health facilities were categorized into optimal-implementation and suboptimal-implementation groups. Barriers and facilitators of the MCH-HB implementation was analyzed via semi-structured interviews to health workers in optimal-implementation and suboptimal-implementation health facilities respectively. The data regarding implementation status were collected using a combination of cross-sectional surveys among health facilities and health workers that took part in the MCH-HB RCT and secondary data sources such as the RCT data and RCT project materials. A structured questionnaire and a semi-structured questionnaire were also conducted among municipality health officers to evaluate municipality-level implementation, barriers, and facilitators of the MCH-HB. The protocol for MCH-HB RCT and this study have been published elsewhere [18, 25].

### Study participants and data source

This study used three data sources. First, to assess the implementation status of the MCH-HB program, a cross-sectional survey was conducted targeting all health facilities in the intervention arm and all five municipality health offices (Supplementary material 1, 2). A quantitative survey to evaluate health workers’ skills and knowledge was conducted among convenient subsamples of health workers in all health facilities (Supplementary material 3). The eligible samples included health workers responsible for the MCH-HB and those in non-management positions. One sample from each category was selected. Data collection for quantitative surveys was conducted by research assistants. Second, to identify the barriers to and facilitators of the implementation of the MCH-HB program, semi-structured interviews were conducted among health workers at convenient subsamples of health facilities and among all municipality health officers responsible for the program. All semi-structured interviews and focus group discussions were facilitated by a group of research assistants using Portuguese as the official language. Third, data from the MCH-HB RCT and operational records at the health facility were used as secondary data sources. The sample size was not determined; however, the health facility survey and health worker survey targeted all health facilities to reduce the health facilities’ sampling bias. The sample size of the interviews was determined based on practical considerations. Data were collected between October and November 2020 and the details are described in the protocol [25].

### Amendment of the protocol

This study originally planned to conduct semi-structured interviews and focus group discussions among 25 health facilities, which was hampered by the COVID-19 control measures. Short semi-structured interviews (N = 155) of approximately 20 min were conducted at 85 health facilities.

### Outcomes

#### Evaluation of MCH-HB’s implementation status

The reach, effectiveness, adoption, implementation, and maintenance (RE-AIM) framework was used to assess the program’s implementation status [26]. RE-AIM is a framework for evaluating the implementation of a target intervention. As “effectiveness” was evaluated in the MCH-HB RCT, the other four constructs were evaluated in this study.

Among the three components of the MCH-HB program, distribution was evaluated in the “reach” dimension. The training of health workers and community sensitization and mobilization were mainly evaluated in the “adoption,” “implementation,” and “maintenance” dimensions.

“Reach” refers to the extent an intervention reaches its target population. Coverage of the MCH-HB at health facilities among new antenatal care service users in September 2020 and coverage among all pregnant women in the municipality or region during the study period were assessed. The target coverage at the health facility level was set at 95% in order to achieve a desirable community coverage of HBR (90%) at the municipality level (under the condition that the health facility utilization rate for receiving antenatal care services still has room for improvement [27]. Coverage of the MCH-HB among all pregnant women in the municipality during the study period was defined as the proportion of the total number of MCH-HB distributed during the study period to the number of all pregnant women in the municipality or region during the study period officially projected using the census data from 2014 [24].

“Adoption” refers to the adoption of the components of the MCH-HB program at the health facility and municipality health office levels. At the health facility level, participation in the training of trainers, intra-facility training, utilization of inventory management sheets, and the provision of mothers’ classes were assessed. At the municipality health office level, the provision of community sensitization or mobilization events and the supervision of health facilities were assessed.

In the “implementation” dimension, implementation fidelity at the health facility level was assessed. The fidelity of the training of health workers was evaluated by two factors — the retention of MCH-HB among MCH-HB RCT participants (MCH-HB retention) and an appropriate description of the child’s birth weight among MCH-HB RCT facility-delivered participants (MCH-HB utilization) [27]. Filling the birthweight of the babies in the MCH-HB was considered as a high priority in the filling of HBRs. The target levels for MCH-HB retention and MCH-HB utilization were pre-defined at 90% and 80%, respectively, according to the previous study and expert opinion [27]. Other fidelity indicators included the stockout of the MCH-HB during the MCH-HB RCT and theme rotation of the mothers’ classes.

“Maintenance” refers to factors that influence the sustainability of the MCH-HB program at a health facility. At the health facility, the existence of a definite person responsible for the intra-facility training after the MCH-HB RCT, the skills and knowledge necessary for the appropriate operation of the MCH-HB program, and the subjective burden of the program were assessed. The same exam used during the trainers’ training was used to evaluate their skills and knowledge. The same target score was set for health workers in charge of the program (70/100 points). A lower target score was set for health workers in non-management positions (60/100 points). The subjective burden was evaluated using a 5-point Likert scale. The introduction of the MCH-HB program at new health facilities established during the MCH-HB RCT period was assessed as an indicator of maintenance at the municipality level. The definitions and targets of the 14 implementation indicators are listed in Table 2.

#### Identification of the barriers to and facilitators of the MCH-HB implementation

The key interview questions were developed based on the Consolidated Framework for Implementation Research (CFIR), a widely used framework to understand the barriers to and facilitators of a target intervention’s implementation [28, 29]. The CFIR has five domains: (1) intervention characteristics, (2) outer setting, (3) inner setting, (4) characteristics of individuals, and (5) process. The facilitators used a few main questions for each CFIR domain—the key questions addressed the difference between the MCH-HB and conventional tools for the intervention characteristics domain, external factors influencing the implementation of the outer setting domain, organizational features influencing the implementation of the inner setting domain, health workers’ ability to utilize the MCH-HB as it is designed for the characteristics of individuals domains, and the feasibility of the plan and problems in execution for the process domain.

### Data analysis

#### Evaluation of the MCH-HB’s implementation status

The implementation variables were descriptively analyzed by the sub-categories of health facilities, such as facility location and health facility level. The missing values were not computed.

At the health facility level, the overall implementation status was evaluated using 14 health facility implementation variables. Continuous variables were converted into binary variables using the pre-defined target level as a threshold. The total score of implementation status, which is the overall implementation score, ranges from 0–14. The target score was pre-defined at 9 out of 14, or a 65% achievement rate, in cases where there were missing values based on expert opinion [25]. Health facilities that reached the target score were categorized as “optimal-implementation facilities” and those that did not reach the target score were categorized as “suboptimal-implementation facilities.”

#### Identification of the barriers to and facilitators of the MCH-HB’s implementation

The transcribed text in Portuguese was corrected to a literary style, where necessary, by a native-language speaking research collaborator. The corrected text was machine-translated into English using DeepL (DeepL GmbH, Cologne, Germany). Ambiguity arising from the machine translation was resolved by the collaborator. Content analysis was conducted using the corrected text.

Due to the protocol amendment, shorter (than originally planned) interviews were obtained from more facilities. As the variability of the quality of the interviews was large, at first, the coding frame was developed among well-spoken subsamples of the interviews until the coding frame reached theoretical saturation. This process was conducted by two researchers’ (AA, AKB) independent coding. Disagreements were resolved by discussions with a third researcher (KT). Subsequently, the coding frame was approved by a group of experts who knew the contexts and the project well (KM, MK, TS). The rest of the interviews were coded by one researcher (AKB) using the coding frame to determine the prevalence of each code in the entire sample.

At the beginning of the process, the translated transcriptions from interviews were divided into two groups: optimal implementation and suboptimal implementation by using the facility’s overall implementation score. Facilitators were coded among the interviews from optimal implementation facilities, while the barriers were from the suboptimal implementation facilities. The obtained coding frame was considered for its corresponding CFIR domains and constructs at the code level by discussion among researchers (AA, AKB, KT, KM). The interviews with the municipality officers were coded using the same coding frame. Additional facilitators and barriers were obtained from the municipality officers’ perspectives.

## Results

For this study, the health facility survey was conducted among 88 facilities (98.9% of the total health facilities participated in the MCH-HB RCT); the health worker survey was conducted among a total of 216 health workers from 87 health facilities (97.8% of the total health facilities) and 155 interviews were conducted with health workers at 85 health facilities (95.5% of the total health facilities), as shown in Table 1. Some facilities did not participate in the health facility survey or health worker survey due to logistical reasons. Municipality health officers of all five municipalities in the intervention group answered the structured questionnaire, and a total of nine municipality health officers answered a semi-structured interview.

**Table 1:** Summary of data collection

Health facility type	Health facility survey n (%)	Health worker survey^a^			Interview^a^ n
		All n	Management position n	Non-management position n	
**All**	88 (98.9%)	216 (87 facilities, 97.8%)	108 (86 facilities, 96.6%)	108 (62 facilities, 69.7%)	155 (85 facilities, 95.5%)
**Facility location type**					
**Urban**	19 (100%)	70 (19 facilities, 100%)	32 (19 facilities, 100%)	38 (16 facilities, 84.2%)	43 (19 facilities, 100%)
**Rural**	69 (98.6%)	146 (68 facilities, 97.1%)	76 (67 facilities, 95.7%)	70 (46 facilities, 65.7%)	112 (66facilities, 94.3%)
**Facility level**					
**Hospital**	6 (100%)	29 (6 facilities, 100%)	14 (6 facilities, 100%)	15 (5 facilities, 83.3%)	14 (6 facilities, 100%)
**Health center**	27 (96.4%)	68 (27 facilities, 96.4%)	35 (27 facilities, 96.4%)	33 (19 facilities, 67.9%)	48 (26 facilities, 92.9%)
**Health post**	55 (100%)	119 (54 facilities, 98.2%)	59 (53 facilities, 96.4%)	60 (38 facilities, 69.1%)	93 (53 facilities, 96.4%)

^a^The number of health facilities from which the survey/interview was conducted and its coverage is described in the parenthesis

### Evaluation of MCH-HB’s implementation status

The indicators for “reach” and “adoption” were found to be relatively high, except for the regular delivery of the mothers’ classes. The indicators for the implementation fidelity were at a relatively low level, especially that for the training of health workers and mothers’ class theme rotation. The MCH-HB retention target was achieved at 48.8% of the health facilities and the MCH-HB utilization at 32.5%, while the mothers’ class theme rotation was at 51.1%. The indicators for maintenance other than the health workers’ subjective burden of the use of MCH-HB was relatively high. The proportion of health workers who answered that the subjective burden was low or very low was less than 10% for both health workers responsible for the MCH-HB and in non-management positions as per Table 2. The summary of the implementation indicators by location and facility type is presented in Supplementary Table 1.

**Table 2:** Facility-based implementation indicators

Indicator	Definition	Target	Not achieved	Achieved	NA	Achievement rate	Data source
**Reach**							
**MCH-HB coverage**	% MCH-HB distribution among new visitors to antenatal/ delivery/ postnatal care services	95%	15	71	3	82.6%	Health facility survey
**Adoption**							
**Training**	Participation in the training of trainers	Yes	5	84	0	94.4%	Project operational records
	Holding an intra-facility training	Yes	10	78	1	88.6%	Health facility survey
**Inventory management**	Use of inventory management logbook	Yes	13	75	1	85.2%	Health facility survey
**Mothers' class**	Holding mothers' classes every week	Yes	35	53	1	60.2%	Health facility survey
**Implementation**							
**MCH-HB retention**	% MCH-HB holders at the end of trial among MCH-HB receivers	90%	43	41	5	48.8%	Baseline survey, endline survey
**MCH-HB utilization**	% Appropriate birth weight description among MCH-HB receivers	80%	54	26	9	32.5%	Endline survey
**Inventory management**	Stock-out	No	11	77	1	87.5%	Health facility survey
**Mothers' class**	Holding mothers' class according to the instruction on themes	Yes	43	45	1	51.1%	Health facility survey
**Maintenance**							
**Intra-facility training**	Definite person in charge of intra-facility training after the trial	Yes	21	67	1	76.1%	Health facility survey
**Skills and knowledge**	A score of a responsible staff member above the required level	70/100	25	61	3	70.9%	Health facility survey
	A median score of staff members above the required level	60/100	12	50	27	80.6%	Health facility survey
**Subjective burden**	Subjective burden of a responsible staff member being "low" or "very low"	Yes	84	2	3	2.3%	Health facility survey
	% Subjective burden of staff members being "low" or "very low"	50%	57	5	27	8.1%	Health facility survey

**Fig. 1:**
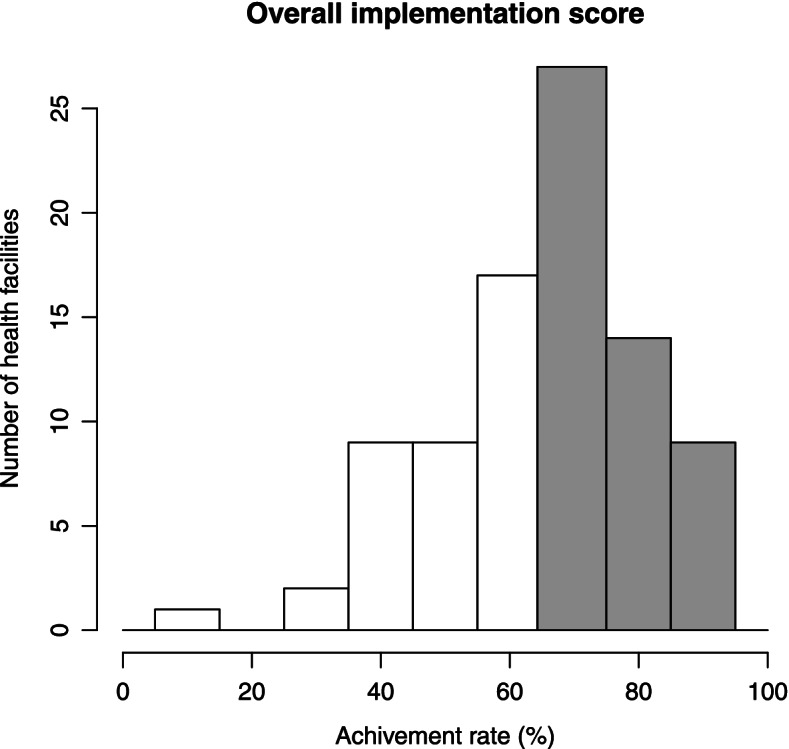
Distribution of the overall implementation score. The gray shade indicates optimal-implementation group defined by the achievement of the overall implementation target (9–14 indicates 64.3% or a 65% achievement rate in case there were missing values)

The overall implementation status was evaluated according to a pre-defined threshold. The mean achievement rate was 63.3%, and 50 health facilities achieved the overall implementation target and were categorized as optimal-implementation facilities (56.8%). Thirty-eight health facilities did not achieve the target and were categorized as suboptimal-implementation facilities (43.2%) (Fig. 1). More health facilities in urban locations (urban 68.4%, rural 53.6%) and at higher facility levels demonstrated better achievement rates (hospital 83.3%, health center 59.3%, health post 52.7%).

The municipality implementation indicators were also assessed. The MCH-HB coverage at the municipality level was 35.3%–83.1% and 53.6% among all five municipalities. Community sensitization or mobilization activity using the MCH-HB material was conducted in one out of five municipalities, and the municipality delivered for 86 times. During the study period, 77.3% health facilities received municipality supervision at least once. Moreover, four new health facilities were established; RCT participants were found in all four health facilities. This indicated that newly established health facilities also implemented the MCH-HB program.

### Identification of the barriers to and facilitators of the MCH-HB’s implementation

Among the barriers and facilitators, categories regarding the MCH-HB itself, the management and supervision of the MCH-HB program, health facility and health workers’ work environment, and users and community were identified. The full structure of the codes and categories, quotes, correspondence to CFIR, and its prevalence among the interviews are summarized in the Supplementary Tables 2 and 3.

#### Barriers to MCH-HB’s implementation

The qualitative analysis of 14 interviews from 14 suboptimal-implementation facilities (four urban, and ten rural: one hospital, four health centers, nine health posts) identified four upper categories and nine lower categories for barriers. The first upper category was “the MCH-HB complexity”, which included two lower categories “complexity of the MCH-HB for health workers” and “complexity of the MCH-HB for users.” The second upper category was “inadequate management and supervision of the MCH-HB in the health facility.” This included two lower categories: “inadequate training for health workers” and “incompetence of health workers in the MCH-HB.” The third upper category was “health facilities' environment,” which included three lower categories: (1) “human resource insufficiency,” (2) “poor learning environment,” and (3) “shortage of resources within the health facility.” The fourth upper category was “user's adherence and factors influencing healthcare use,” which further included two lower categories: “poor user adherence to health services” and “factors hindering healthcare use.” The entire suboptimal-implementation coding demonstrated that the most prevalent barrier was “inadequate training for health workers” (65.7%), followed by “complexity of the MCH-HB for health workers” (62.7%), and “shortage of resources within the health facility” (62.7%) (Supplementary Table 2).

#### Facilitators of the MCH-HB implementation

The qualitative analysis of the 13 interviews from the 13 optimal-implementation facilities (three hospitals, three health centers, and seven health posts; five urban and eight rural) identified four upper categories and 12 lower categories for facilitators. The first upper category was “the MCH-HB advantages,” which included two lower categories: “the MCH-HB benefit for health workers' practice” and “the MCH-HB content advantage.” The second upper category was “the appropriate MCH-HB management and supervision at health facilities,” which included six lower categories: (1) “adequate training on the MCH-HB for health workers,” (2) “effective municipality supervision on the MCH-HB in health facilities,” (3) “health workers’ high competency,” (4) “leadership in health facilities,” (5) “appropriate stock management of the equipment related to the MCH-HB,” and (6) “existence of evaluation and the feedback system in HFs.” The third upper category was “health facilities’ and health workers’ positive attitudes toward work,” which included two lower categories: “health workers’ acceptance to the MCH-HB” and “health workers’ positive learning and working attitudes.” The fourth upper category was “users’ acceptance and community involvement,” which included two lower categories: “community involvement for the MCH-HB program” and “user's acceptance and adherence to the MCH-HB.” The entire optimal-implementation text coding demonstrated that the most prevalent facilitator was “the MCH-HB content advantage” (96.6%), followed by “high competency of health workers” (90.9%) and “health workers’ acceptance toward the MCH-HB” (85.2%) (Supplementary Table 3).

#### Barriers and facilitators from municipality health officers

Two additional barriers were identified from the nine interviews with municipality health officers: (1) human resource insufficiency and shortage in resources and budget at the municipality level; and (2) a lack of transportation for supervision and community activities. Three additional facilitators were identified: (1) adequate human resources at the municipality level; (2) high competency of health officers; and (3) support from the provincial level.

### Contextual changes

Due to the COVID-19 pandemic, a state of emergency was issued in Angola from March to May 2020, and a state of public calamity was issued subsequently, which continues until now. The government requested citizens to not travel, unless necessary. The interview analysis demonstrated that the COVID-19 pandemic hindered the users’ healthcare utilization. Municipality officers’ out-reach activities were also restricted during the pandemic.

### Harms and unintended effects

No harms or unintended effects were observed.

## Discussion

This study aimed to evaluate the MCH-HB’s implementation status among the intervention-group’s health facilities and to identify the barriers to and facilitators of the MCH-HB’s implementation. This study approached 88 health facilities for health facility surveys, 216 health workers for health workers’ surveys, and conducted 155 interviews. The achievement rate was high among the “reach” and “adoption” dimension indicators but was low in the “implementation” dimension indicators and burden indicators in the “maintenance” dimension. The evaluation of the overall implementation status demonstrated that 50 health facilities (56.8%) achieved the implementation targets. The target was achieved by more health facilities in urban than rural areas (68.4% vs. 53.6%). Further, more health facilities of higher facility types achieved the target (hospital 83.3%, health center 59.3%, health post 52.7%). On analyzing the interviews, the barriers to and facilitators of the MCH-HB’s implementation were identified, which were composed of four upper categories: each about the MCH-HB itself, the management and supervision of the program, health facility and health workers’ work environment, and users and community. To the authors’ knowledge, this is the first study that examined the implementation status of the MCH-HB and the barriers and facilitators for its implementation using theoretical framework for implementation research. Comparison with previous studies.

Compared to previous studies in the African region, the retention rate and estimated reach of the MCH-HB among all pregnant women were relatively low [27]. However, Angola is one of the countries with biggest challenges in maternal and child health [3]. In addition, a previous study suggested that the utilization and retention of HBRs and the strengthening of national immunization programs is required in many countries [27]. Prior this study, the WHO guidelines described HBRs’ potential challenges, such as overburdening health workers, inadequate training of health workers, and poor maternal acceptance or adherence to HBRs [9]. This study’s interview analysis results demarcated these issues as barriers to the MCH-HB’s implementation in Angola. The guideline noted implementation considerations such as culturally adopted content and design of the MCH-HB, adequate training of health workers and supervision, and users’ acceptance, similar to our demarcation of facilitators [20]. The guidelines also posed a specific implementation question about out-of-pocket payments for HBRs. In our study, free distribution was identified as a facilitator at a code level (Supplementary Table 3). However, this may depend on the context of MCH-HB implementation. A previously conducted study examining the effectiveness of MCH-HB in Cambodia noted the complexity of the educational content for users, their poor acceptance, and cultural and religious beliefs that contradict the MCH-HB’s contents as barriers and efforts to attract users and to enhance their understanding, such as the use of pictures as facilitators [11]. These factors were also identified at a code level in our study, as indicated by the Supplementary Table 2 and 3. Another MCH-HB effectiveness study in Mongolia demonstrated that women with lower socioeconomic status were less likely to benefit from the program [10]. We also identified users’ economic and physical impediments to healthcare use as a barrier to the MCH-HB’s implementation. Due to the similarity between the past considerations and the barriers and facilitators identified in this study, our results are considered to have high generalizability.

### Implications for practice—possible implementation strategies

Prior to this study, education for health workers to ensure appropriate use of the MCH-HB and community sensitization or mobilization to ensure the uptake of MCH-HB were treated as intervention components. However, these components may also be considered as implementation strategies to support health workers in delivering MCH-HB services to users according to the theoretical model of implementation science. The results of this study have several implications for implementation strategies.

Approximately half of the health facilities did not achieve the pre-defined implementation target; this was especially difficult in rural areas and among lower-level health facilities. This was because ensuring the implementation fidelity of key intervention components (educating users to keep the MCH-HB, filling the MCH-HB, delivering mothers’ classes with various themes, etc.) was difficult. The mere adoption of the key components (participating in the training of trainers, delivering intra-facility training, delivering mothers’ classes regularly, etc.) was achieved at a higher level in Angola. Hence, the improvement in implementation fidelity is key to achieving better implementation of the MCH-HB and in contributing to the improvement in the CoC in Angola. To improve implementation fidelity, the provision of training for health workers is the core implementation strategy. Furthermore, factors related to the adequacy of training and municipality supervision were identified in both barriers and facilitators. Training of trainers, intra-facility training, and local governments’ supervision were already employed in our study to ensure education for health workers. The barriers and facilitators identified in the interview analysis suggested the need to further strengthen the training and supervision of current health workers, such as the provision of training to all health workers in the facility, refresher training and continuous training, and frequent municipality supervision.

Furthermore, factors related to users’ acceptance of MCH-HB and their adherence to MNCH services were identified as both barriers and facilitators. The delivery of community sensitization events, involvement of community stakeholders, such as religious leaders and village leaders, and coordination with other health promotion activities were suggested as core implementation strategies in the interviews. A previous study in Bangladesh reported the potential advantage of mobile platform to increase women’s adherence to MNCH services [30]. Use of mobile technologies needs to be carefully considered once mobile technologies becomes widely available.

Finally, our study demonstrated the disparity in the implementation status between urban-area-based facilities and rural-area-based facilities and between lower-level and higher-level facilities. Lower-level facilities and rural area-based facilities require a more comprehensive and intensive implementation strategy, considering the demonstrated disparity. For the delivery of municipality supervision and community sensitization or mobilization events, ensuring transportation is crucial.

Health interventions sometimes include implementation strategies as their intervention components. Recognizing implementation strategies encapsulated in the intervention enables a better evaluation of its effectiveness and implementation.

### Application of implementation research frameworks in global health context

Despite its simple structure, the RE-AIM framework enabled better understanding of the MCH-HB impact. Inclusion of implementation variables based on frameworks such as the RE-AIM framework is recommended for future research due to its easiness and informativeness. The CFIR framework is a comprehensive framework and takes some time to complete evaluation using it. However, even a small number of interviews or group discussions using the CFIR before and/or after the implementation would be worth conducting. Reaching appropriate targets who can analyze the settings and verbalize their opinions, and ensuring appropriate facilitators are crucial to maximize the benefits of the CFIR framework in global health contexts.

### Limitations

This study has several limitations. First, the implementation indicators and their target achievement levels were defined by our research team and experts of the MCH-HB. There may have been some missed informative indicators, despite the selection of the indicators being based on the MCH-HB and RE-AIM frameworks’ core components. However, the overall implementation score demonstrated reasonable variability. The overall implementation target achievement rate demonstrated a reasonable difference between the types of health facilities and the location of the health facilities. Further research focusing on implementation is necessary to better evaluate the MCH-HB’s implementation.

Due to the COVID-19 control measures, interviews were time-constrained and could not obtain in-depth testimonies. Moreover, the interviews were transcribed, modified into article-style, and machine translated. This process may have diminished some detailed information from the texts.

This study examined health facilities, health workers, and municipal health officers and did not examine users’ perceptions. Despite questions on the users’ perceptions being asked in the interviews with health workers and municipality health officers, barriers and facilitators from the users’ perspectives were not sufficiently captured.

## Conclusion

Our study demonstrated the need to further improve the MCH-HB program’s implementation to obtain its full benefits in Angola. The key to improving this was implementation fidelity, such as MCH-HB retention and utilization. The qualitative analysis comprehensively demonstrated the barriers to and facilitators of the implementation of the MCH-HB. The identified barriers and facilitators replicated many factors that were assumed but not scientifically demonstrated in previous HBRs’ studies. Continuous training for all health workers, frequent and close supervision by local governments, and community sensitization or mobilization were considered reasonable implementation strategies.

## Supplementary Information

**Additional file 1. Supplementary material 1. **Survey to health facilities.

**Additional file 2. Supplementary material 2.** Survey to municipality health bureaus.

**Additional file 3. Supplementary material 3.  **Health workers’ survey.

**Additional file 4: Supplementary Table 1.** Barriers to the MCH-HB implementation.

**Additional file 5: Supplementary Table 2.** Facilitators of MCH-HB implementation.

**Additional file 6: Supplementary Table 3. **Facility-level implementation indicators by location and facility type.

## Data Availability

The datasets generated and/or analyzed during this study are available from the corresponding author upon reasonable request.

## Abbreviations

CoC: Continuum of care
CFIR: The Consolidated Framework for Implementation Research
HBR: Home-based record
LMICs: Low- and middle-income countries
MNCH: Maternal, neonatal, and child health
MCH-HB: Maternal and child health handbook
RCT: Randomized controlled trial
RE-AIM: Reach, effectiveness, adoption, implementation, and maintenance
SDGs: Sustainable Development Goals
WHO: World Health Organization

## References

[1] United Nations. Sustainable development goals. https://www.un.org/sustainabledevelopment/sustainable-development-goals/. Accessed 17 Jan 2022

[2] World Health Organization. Trends in maternal mortality: 2000 to 2017: estimates by WHO, UNICEF, UNFPA, World Bank Group and the United Nations population division.; 2019. https://apps.who.int/iris/bitstream/handle/10665/327596/WHO-RHR-19.23-eng.pdf?sequence=13&isAllowed=y. Accessed 1 Jul 2022.

[3] UN. IGME. Levels and trends in child mortality 2020; 2020. https://www.unicef.org/reports/levels-and-trends-child-mortality-report-2020. Accessed 17 Jan 2022

[4] Kikuchi K, Okawa S, Zamawe CO, Shibanuma A, Nanishi K, Iwamoto A (2016). Effectiveness of continuum of care-linking pre-pregnancy care and pregnancy care to improve neonatal and perinatal mortality: A systematic review and meta-analysis. PLoS ONE.

[5] Kerber KJ, de Graft-Johnson JE, Bhutta ZA, Okong P, Starrs A, Lawn JE (2007). Continuum of care for maternal, newborn, and child health: from slogan to service delivery. Lancet.

[6] de Graft-Johnson J, Kerber K, Tinker A, Otchere S, Narayanan I, Shoo R, et al. The maternal, newborn and child health continuum of care. Oppor Afr’s Newborns. 2006:23–36

[7] Osaki K, Aiga H (2016). What is maternal and child health handbook?.

[8] Osaki K, Aiga H (2019). Adapting home-based records for maternal and child health to users’ capacities. Bull World Health Organ.

[9] World Health Organization. WHO recommendations on home-based records for maternal, newborn and child health. World Health Organization. 2018. https://www.who.int/publications/i/item/9789241550352. Accessed 17 Jan 2022. 30325618

[10] Mori R, Yonemoto N, Noma H, Ochirbat T, Barber E, Soyolgerel G (2015). The maternal and child health (MCH) handbook in Mongolia: a cluster-randomized, controlled trial. PLoS ONE.

[11] Yanagisawa S, Soyano A, Igarashi H, Ura M, Nakamura Y (2015). Effect of a maternal and child health handbook on maternal knowledge and behaviour: a community-based controlled trial in rural Cambodia. Health Policy Plan.

[12] Osaki K, Hattori T, Toda A, Mulati E, Hermawan L, Pritasari K (2019). Maternal and Child Health Handbook use for maternal and child care: a cluster randomized controlled study in rural Java, Indonesia. J Public Health (Oxf).

[13] Aiga H, Nguyen VD, Nguyen CD, Nguyen TT, Nguyen LT (2016). Fragmented implementation of maternal and child health home-based records in Vietnam: need for integration. Glob Health Action.

[14] Bhuiyan SU, Nakamura Y. Continuity of maternal, neonatal and child health care through MCH handbook for ensuring the quality of life. In: Bhuiyan SU, Nakamura Y, editors. 2008 MCH handbook conference report. Osaka University and HANDS; 2009.

[15] Osaki K, Hattori T, Kosen S (2013). The role of home-based records in the establishment of a continuum of care for mothers, newborns, and children in Indonesia. Glob Health Action.

[16] Carandang RR, Sakamoto JL, Kunieda MK, Shibanuma A, Yarotskaya E, Basargina M, et al. Roles of the maternal and child health handbook and other home-based records on newborn and child health: A systematic review. Int J Environ Res Public Health. 2021;18. 10.3390/ijerph18147463 10.3390/ijerph18147463 PMC8306696 34299924

[17] Nakamura Y. The role of maternal and child health (MCH) handbook in the era of sustainable development goals (SDGs). J Glob Health Sci. 2019;1. 10.35500/jghs.2019.1.e24

[18] Balogun OO, Tomo CK, Mochida K, Mikami M, da Rosa VH, Neves I (2020). Impact of the Maternal and Child Health handbook in Angola for improving continuum of care and other maternal and child health indicators: study protocol for a cluster randomised controlled trial. Trials.

[19] Yapa HM, Bärnighausen T (2018). Implementation science in resource-poor countries and communities. Implement Sci.

[20] Mahadevan S, Broaddus-Shea ET (2020). How should home-based maternal and child health records be implemented? A global framework analysis. Glob Health Sci Pract.

[21] World Bank. The World Bank open data. https://data.worldbank.org/. Accessed 17 Jan 2022

[22] WHO. Quadro de Parceria entre o Governo de Angola e o Sistema das Nações Unidas (UNPAF); 2015–2019. https://www.afro.who.int/publications/quadro-de-parceria-entre-o-governo-de-angola-e-o-sistema-das-nacoes-unidas-unpaf-2015. Accessed 17 Jan 2022

[23] Green A (2017). Health in Angola in the wake of the presidential election. Lancet.

[24] Instituto Nacional de Estatística. Projecção da população província de Benguela; 2014. https://www.ine.gov.ao/Arquivos/arquivosCarregados//Carregados/Publicacao_637586904770085848.pdf. Accessed 17 Jan 2022. Luanda: Instituto Nacional de Estatística. p. 2016

[25] Aoki A, Mochida K, Kuramata M, Sadamori T, Freitas HR, da Cunha JD (2021). The maternal and child health handbook for improving the continuum of care and other maternal and child health indicators in Angola: an implementation study protocol. Front Glob Womens Health.

[26] Glasgow RE, Vogt TM, Boles SM (1999). Evaluating the public health impact of health promotion interventions: the RE-AIM framework. Am J Public Health.

[27] Brown DW, Gacic-Dobo M (2015). Home-based record prevalence among children aged 12–23 months from 180 demographic and health surveys. Vaccine.

[28] Damschroder LJ, Aron DC, Keith RE, Kirsh SR, Alexander JA, Lowery JC (2009). Fostering implementation of health services research findings into practice: a consolidated framework for advancing implementation science. Implement Sci.

[29] CFIR Research Team. Center for Clinical Management Research. Consolidated framework for implementation research; 2022. https://cfirguide.org/. Accessed 17 Jan 2022

[30] Gai Tobe R, Haque SE, Mubassara S, Rahman R, Ikegami K, Mori R (2022). Maternal and child health handbook to improve continuum of maternal and child care in rural Bangladesh: Findings of a cluster randomized controlled trial. PLoS ONE.

