# Supplementary material for: The RE-AIM framework-based evaluation of the implementation of the Maternal and Child Health Handbook program in Angola: a mixed methods study
Source: BMC Health Serv Res. 2022 Aug 22;22:1071. doi: 10.1186/s12913-022-08454-9 (PMC9395902; doi:10.1186/s12913-022-08454-9)
Supplement: Supplementary file 1 — Additional file 1. Supplementary material 1. Survey to health facilities. [file 12913_2022_8454_MOESM1_ESM.docx]

**Survey to health facilities**

|  | Question item | Answer | |
| --- | --- | --- | --- |
| 1 | Did your facility had a training of MCH-HB for healthcare providers? | 1: Yes | 2: No |
| 2 | Does your facility use the inventory management logbook? | 1: Yes | 2: No |
| 3 | (If the facility uses the inventory management logbook, check whether it is appropriately used) | 1: Appropriate | 2: Not appropriate |
| 4 | Did your facility experienced stock out of MCH-HB between April 2019 and May 2020? | 1: Yes | 2: No |
| 5 | Did your facility provide a mothers' class using "flipchart" this week? | 1: Yes | 2: No |
| 6 | (If mothers' class was provided this week) What was the theme of mothers' class this week? | Theme: | |
| 7 | What was the theme of mothers' class two weeks ago? | Theme: | |
| 8 | Does your facility appoint a person in charge of training of MCH-HB after the trial? | 1: Yes | 2: No |
| 9 | (If your facility has a person in charge of training) Who is the person? | Person in charge: | |
| 10 | Did your facility had a supervision of MCH-HB by a municipality focal person between April 2019 and May 2020? A supervision of municipality focal person means a visit of municipality focal person without accompanying foreign staff. | 1: Yes | 2: No |
| 11 | How many new patients visited ANC service in September 2020? | Number: | |
| 12 | How many MCH-HB has your facility distributed in September2020? | Number: | |
