# Supplementary material for: The RE-AIM framework-based evaluation of the implementation of the Maternal and Child Health Handbook program in Angola: a mixed methods study
Source: BMC Health Serv Res. 2022 Aug 22;22:1071. doi: 10.1186/s12913-022-08454-9 (PMC9395902; doi:10.1186/s12913-022-08454-9)
Supplement: Supplementary file 2 — Additional file 2. Supplementary material 2. Survey to municipality health bureaus. [file 12913_2022_8454_MOESM2_ESM.docx]

**Survey to municipality health bureaus**

|  | Question item | Answer | |
| --- | --- | --- | --- |
| 1 | Did you hold community sensitization/ mobilization activities using "flipchart"? | 1: Yes | 2: No |
| 2 | (If you provided the activities) How many times did you hold the activities during between April 2019 and May 2020? | Total number: | |
