# Supplementary material for: The RE-AIM framework-based evaluation of the implementation of the Maternal and Child Health Handbook program in Angola: a mixed methods study
Source: BMC Health Serv Res. 2022 Aug 22;22:1071. doi: 10.1186/s12913-022-08454-9 (PMC9395902; doi:10.1186/s12913-022-08454-9)
Supplement: Supplementary file 3 — Additional file 3. Supplementary material 3. Health workers’ survey. [file 12913_2022_8454_MOESM3_ESM.doc]

**Health workers’ survey**

This questionnaire asks the health workers of the health units about the state of implementation of the MCH-HB in the health facility.

**Municipality:**

**Health facility:**

Circle the option applicable for you.

**Your working Area / Section:**

1. ANC
2. Delivery
3. Vaccination
4. Child healthcare
5. Others

**Your position:**

1. Management position
2. Non-management position

**Section1. Skills and knowledge**

Circle the statement with the correct answer.

1. **The objective of the Maternal and Child Health Handbook (MCHH) is to:**
2. Promote home deliveries.
3. Maintain MCHH in the health facility by healthcare providers to monitor maternal-infant health conditions.
4. Ensure health records from gestation until the child reaches 5 years of age.
5. Give the most important attention to the healthcare providers.
6. **The healthcare providers' explanation about MCHH is given to the mother as follows:**
7. Please read the MCHH on your own.
8. The MCHH paper will end when the child is 5 years old and can go to bed.
9. Important content is written in the MCHH, please do not show it to anyone.
10. Parents should bring it with them every time they go to consultations.
11. **Who should receive the MCHH?**
12. All mothers / children who get the vaccine during the vaccination campaign.
13. Pregnant woman coming for ANC, not only for the first, but also 2nd, 3rd, 4th time etc. who had not yet received the "Maternal and Child Health Handbook".
14. The healthcare providers should not give the 2nd MCHH to the mother, even when twins are born.
15. Mothers who gave birth at home and not at health facilities.
16. **Choose the correct sentence about ANC.**
17. ANC is required after 5 months of pregnancy.
18. Four ANC visits are required before giving birth.
19. Integrated care throughout pregnancy.
20. It is important to come alone without the accompaniment of family members.
21. **What is the expected date of delivery (EDD) for a pregnant woman who last menstruated on November 25, 2018?**
22. 2 of September, 2019
23. 2 of October, 2019
24. 25 of September, 2019
25. 25 of October, 2019
26. **Today is July 11. What is the likely gestational age (GA) of a MULTIPARA who last menstruated on January 1, 2018?**
27. 25 weeks
28. 26 weeks
29. 27 weeks
30. 28 weeks

1. **On January 1, 2019 a pregnant woman came in for her ANC appointment; we determined that her fundal height was 29 cm.**

**What is the gestational age (GA)?**

1. 40 weeks (40th week)
2. 37 weeks (37th week)

| **GA in weeks** | **Fundal hight** |
| --- | --- |
| **20 weeks** | 18 cm |
| **22 weeks** | 20 cm |
| **24 to 25 weeks** | 22 cm |
| **27 Sweeks** | 24 cm |
| **29 weeks** | 25 cm |
| **31 weeks** | 27 cm |
| **33 weeks** | 29 cm |
| **37 weeks** | 32 cm |
| **40 weeks** | 34 to 35 cm |

1. 31 weeks (31st week )
2. 33 weeks (33rd week)
3. **On January 1, 2019 a pregnant woman came in for her ANC appointment; we determined that her fundal height was 29 cm.**

**What is the expected date of delivery (EDD)?**

1. 19 of February, 2019
2. 8 of October, 2019
3. 14 of May, 2019
4. 25 of October, 2019

1. **Intermittent preventive treatment (IPTp) of malaria in pregnant women can start from 　　　　weeks of gestation:**
2. 10 weeks (10th week)
3. 11 weeks (11th week)
4. 12 weeks (12th week)
5. 13 weeks (13th week)
6. **Intermittent preventive treatment (IPTp) of malaria in pregnant women, at least how many doses should be given:**
7. 2 doses
8. 3 doses
9. 4 doses
10. 5 doses
11. **Every pregnant woman should get at least 　　　　 doses of anti-tetanus vaccine:**
12. 0
13. 1
14. 2
15. 3
16. **What vaccines should be given to a newborn before discharge?**
17. Hepatitis B, polio
18. BCG, polio 0, Penta
19. Polio, BCG, Hepatitis A
20. Polio, BCG, Hepatitis B
21. **At childhealth check-up consultation at different ages:**
22. Assessment of the child's growth curve, physical, vaccination, and development/behavior are very important.
23. In counseling the family, the involvement of the mother is more important than the father.
24. Parents should not be given space to raise doubts and concerns.
25. Issues such as breastfeeding and varied feeding after 3 months are reinforced in counseling.
26. **A child under 6 months of age is considered severely malnourished if he or she has:**
27. Brachial circumference < 150 mm
28. Brachial circumference < 120 mm
29. Brachial circumference < 110 mm
30. Other answers.
31. **The most important advantage of breast milk is:**
32. That it is cheaper.
33. That it contains everything the child needs.
34. That it contains more protein and vitamins.
35. All of them are correct.
36. **Giving porridge or other food to a child under 6 months:**
37. You should not give juice and soda, but you can give kissangua (local fermented juice).
38. It can be harmful because the stomach is not prepared.
39. It is necessary to gain strength and weight.
40. It is necessary to get used to it.
41. **What is the correct method for measuring the height and weight of a child under 2 years of age?**
42. Children older than 6 months should be measured standing.
43. Height is not related to nutrition, so children under 2 years of age do not need to be measured.
44. The scale and altimeter should be positioned on a horizontal table.
45. To prevent children from catching a cold, one should measure with clothes on.
46. **What is the correct interpretation about the growth monitoring curve?**
47. It is important to observe if the child is growing according to the average age, so the individual growth curve is not important.
48. The shape of boys and girls is the same.
49. Above the red line (3) and below the red line (-3) means that the child is doing well.
50. Reflects the health and nutrition conditions of the child attended.
51. **Fill in the chart below. (Weight, Boy)**

| At birth | 3,000g |
| --- | --- |
| 6 weeks | 7.0Kg |
| 1 year | 10.0Kg |
| 3 years | 16.0Kg |


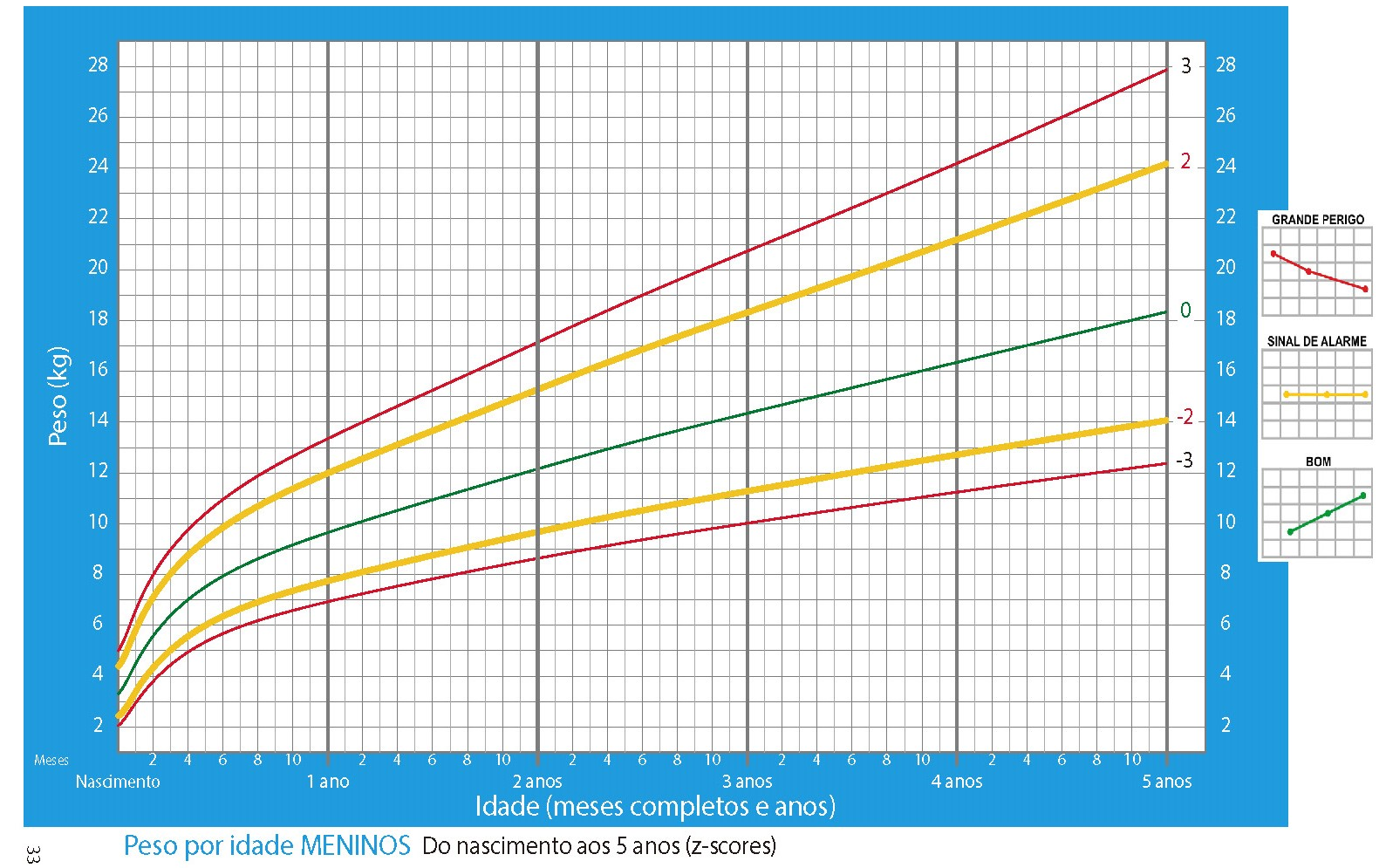


1. **The talks to mothers are effective when:**
2. Done for 30 minutes every day.
3. Using lots of technical terms so mothers learn more.
4. Giving mothers and family members opportunities to ask questions.
5. Holding lectures with the same themes every day so that the mothers don't forget the contents (themes).

**Section2. Burdens**

Circle the option applicable for you.

**How high is the burden of using MCH-HB for you?:**

1. Very high
2. High
3. Not high nor low
4. Low
5. Very low

*Section 1: Question 1-20 are scored 5 points each, and the total score was calculated. The total score ranges from 0 to 100.
