# Supplementary material for: The RE-AIM framework-based evaluation of the implementation of the Maternal and Child Health Handbook program in Angola: a mixed methods study
Source: BMC Health Serv Res. 2022 Aug 22;22:1071. doi: 10.1186/s12913-022-08454-9 (PMC9395902; doi:10.1186/s12913-022-08454-9)
Supplement: Supplementary file 4 — Additional file 4: Supplementary Table 1. Barriers to the MCH-HB implementation. [file 12913_2022_8454_MOESM4_ESM.docx]

**Supplementary Table 1** Barriers to the MCH-HB implementation

| Level | Categories and codes | Quotes | Observations among all interviews | Corresponding CFIR domains/ constructs |
| --- | --- | --- | --- | --- |
| Upper Category | The MCH-HB complexity |  | 64.2% |  |
| Lower Category | Complexity of the MCH-HB for HWs |  | 62.7% |  |
| Code | Complexity in the assessment required for the MCH-HB | we didn't understand and ready, sometimes even mainly when filling out the form, to find the gestational age and the heartbeat of the pre-natal, newborn, fetus, right. (E28) | 17.9% | Intervention characteristics/ complexity |
| Code | Complexity in recording the MCH-HB | Many of our technicians, because they don't fit well with the new notebook, forget to write down all the data, both on weight and height. (E37) | 43.3% |  |
| Code | Complexity in the contents of the MCH-HB | For me give more content. Not only for the mothers and the nurses also need more content about feeding the child. (B3) | 7.5% |  |
| Code | HWs' resistance toward the MCH-HB | Why is the MCH-HB having any merit? -It is not found. (D22) | 9.0% | Intervention characteristics/ relative advantage, complexity, characteristics of individuals/ knowledge and beliefs about the intervention |
| Lower Category | Complexity of the MCH-HB for users |  | 9.0% |  |
| Code | Complexity of the MCH-HB for users | They perceive accurately the vaccine card, they like the MCH-HB, but the perception is a little difficult, some say they understood but they don't understand better the MCH-HB. (D29) | 9.0% | Intervention characteristics/ complexity, outer setting/ patient needs and resources |
| Upper Category | Inadequate management and supervision of the MCH-HB in the HF |  | 80.6% |  |
| Lower Category | Inadequate training for HWs |  | 65.7% |  |
| Code | Inadequate municipality supervision | Did you have any intervention from a focal person from the municipality regarding the MCH-HB? - No intervention, although she accompanied us the way she did, she followed us, but not up to this point. (C12) | 32.8% | Process/ plan, executing, reflecting and evaluating, characteristics of individuals/ other personal attributes |
| Code | No continuous training for HWs within the HF | This colleague as she is new she has never participated in that workshop yet … At the time of the training she was not yet placed. (C20) | 10.4% | Inner setting/ readiness for implementation (available resources), process/ engaging |
| Code | Inadequate intra-facility training at the beginning of MCH-HB program | Those were the ones that gave us training for just one day. Yes, that happened in that delivery room, but it wasn't for all the colleagues. (B9) | 23.9% |  |
| Code | Absence of a responsible person | In your health unit do you have a dignified person responsible for the implementation of the maternal and child health booklet? - We don't have one yet, honestly speaking we don't. (D18) | 25.4% | Inner setting/ readiness for implementation (leadership engagement) |
| Code | The responsible person’s inadequate teaching capacity | Here there is no technician that went through the training and here they never had any training? -Never, wait, when was this training? (B3) | 4.5% | Inner setting/ readiness for implementation (available resources), characteristics of individuals/ other personal attributes |
| Code | Inadequate ToT | When the booklet came we went to the training, but we didn't follow everything. (B5) | 3.0% | Process/ plan, Inner setting/ readiness for implementation (available resources), characteristics of individuals |
| Lower Category | Incompetence of HWs in the MCH-HB |  | 53.7% |  |
| Code | HWs' incompetence regarding the use of the MCH-HB | I would not say that they are confident in the Maternal and Child Health Booklet because they still continue to register some errors in the ANC services. (D4) | 40.3% | Characteristics of individuals/ knowledge and beliefs about the intervention, individual stages of change |
| Code | HWs' incompetence regarding general MNCH services | In childcare and in the vaccination program I still have difficulty because those are areas that I don't have much affinity for. (B11) | 11.9% | Characteristics of individuals/ knowledge and beliefs about the intervention, self-efficacy, individual stages of change |
| Code | Responsible person not functioning | Why haven't you ever had an exchange of experience to improve the use of the MCH-HB? - Who should schedule that type of activities is the head of ANC service. (D11) | 9.0% | Inner setting/ readiness for implementation (leadership engagement) |
| Upper Category | HFs' environment |  | 77.6% |  |
| Lower Category | Human resource insufficiency |  | 35.8% |  |
| Code | Inadequate number of HWs | Because we don't have staff. If we did we could spread the work out. (A5) | 17.9% | Inner setting/ readiness for implementation (available resources) |
| Code | Specific positions not being filled | Here the center so far has twelve people, even so lacking a laboratory technician, that we could not make requests for analysis. (D3) | 6.0% |  |
| Code | HWs being overburdened | But I really see that it is a lot of overload, only in the case of vaccines they forced us so much... there is no way that only two people can work so much. (B22) | 28.4% |  |
| Lower Category | Poor learning environment |  | 20.9% |  |
| Code | Unwillingness to seek help, and to teach each other | The problem is that you have the questions but you don't present. (A7) | 10.4% | Inner setting/ implementation climate (learning climate) |
| Code | No opportunity to exchange ideas to better utilize the MCH-HB | We just have that meeting ..., it's not just about the notebook and we haven't exchanged ideas regarding that. (B24) | 4.5% |  |
| Code | No structured evaluation or feedback system in the HF | I don't know how they evaluate me I can't do self-assessment. (D16) | 10.4% | Inner setting/ goals and feedback, implementation climate (learning climate) |
| Lower Category | Shortage of resources within the HF |  | 62.7% |  |
| Code | Lack of general equipment | We can't deliver and reserve the medication because, like last months, we stayed two months without receiving the medication, ... the output is higher and the delivery is lower. (C12) | 59.7% | Inner setting/ readiness for implementation (available resources) |
| Code | Stockout of the MCH-HB | The most difficult point of the maternal and child health booklet that complicates us is when we don't have it. (C28) | 11.9% | Process/ plan, executing |
| Upper Category | User's adherence and factors influencing healthcare use |  | 73.1% |  |
| Lower Category | Poor user adherence to health services |  | 56.7% |  |
| Code | Users' resistance toward the MCH-HB | Since we entered (to the MCH-HB program) not all notebooks have instructed us because mothers don't show up either. (C5) | 23.9% | Intervention characteristics/ relative advantage, Outer setting/ patient needs and resources |
| Code | Users' poor adherence to the MNCH services | What is most difficult for us are the mothers themselves, because in the regulations it says that a mother should come in the first trimester, but sometimes they don't want to collaborate with us. (A7) | 49.3% | Outer setting/ patient needs and resources |
| Lower Category | Factors hindering healthcare use |  | 55.2% |  |
| Code | Users' low health literacy | The difficulty has always been this, because there are certain data that the colleague analyzes and evaluates due to the mother's lack of knowledge. (E12) | 31.3% | Outer setting/ patient needs and resources |
| Code | Cultural/religious barriers to healthcare use | There are some who comply, there are others with superior orientations back home, the husband forces them to carry heavy things. (B10) | 20.9% |  |
| Code | Physical/economic barriers to healthcare use | In general, not everyone is able to buy a prescription drug, some of them are needy and writing the prescription is a loss for them. (C22) | 28.4% |  |

* Parentheses placed after a quotation indicates the interview from which the quotation was derived. A, B, C, D, E indicate five municipalities each.

CFIR: Consolidated Framework for Implementation Research, HW: Health-workers; MNCH: maternal, neonatal, and child health services; MCH-HB: Maternal and child health handbook; HF: health facilities; ToT: training of trainers.
