# Supplementary material for: The RE-AIM framework-based evaluation of the implementation of the Maternal and Child Health Handbook program in Angola: a mixed methods study
Source: BMC Health Serv Res. 2022 Aug 22;22:1071. doi: 10.1186/s12913-022-08454-9 (PMC9395902; doi:10.1186/s12913-022-08454-9)
Supplement: Supplementary file 5 — Additional file 5: Supplementary Table 2. Facilitators of MCH-HB implementation. [file 12913_2022_8454_MOESM5_ESM.docx]

**Supplementary Table 2** Facilitators of MCH-HB implementation

| Level | Categories and codes | Quotes | Observations among all interviews | Corresponding CFIR domains/ constructs |
| --- | --- | --- | --- | --- |
| Upper Category | The MCH-HB advantages |  | 98.9% |  |
| Lower Category | The MCH-HB benefit for HWs' practice |  | 75.0% |  |
| Code | Helping HWs better detect health problems | (For example) you have the mother weighed 50, and this month she weighed 49, then you will call attention, something is missing there, that is why this MCH-HB has great importance, because it will accompany the mother. (B13) | 22.7% | Intervention characteristics/ relative advantage |
| Code | Helping HWs to better assess users' health status | the notebook is a vehicle or an instructional medium that helps both the pregnant mother and much more in particular the professional in following the maternal child development. (B12) | 60.2% |  |
| Code | Helping HWs to better deliver health education | the MCH-HB helps us as technicians to spread the information and pass the message. (E34) | 25.0% |  |
| Code | Helping HWs to provide better, and efficient MNCH services | Because I think so because this card facilitates a lot in my day-to-day work, like vaccination, weight, perimeter and other things. (C9) | 26.1% |  |
| Lower Category | The MCH-HB content advantage |  | 96.6% |  |
| Code | Integration of the child and mother part and a longer follow-up | I think that these notebooks are important because it is no longer necessary for the mother to carry two cards, because the same one serves for pregnancy, serves for the follow-up of the child at vaccination, and even when the child is sick, the mother can carry this notebook. (D13) | 85.2% | Intervention characteristics/ relative advantage |
| Code | More organized, and clearer contents | The advantage is that this one brings more information and is more explicit, both for health professionals and for the mother and her companions or families. (C14) | 65.9% |  |
| Code | Strengthening family bonding | I think that the merit would be the union of the family. (D7) | 27.3% |  |
| Code | Being distributed free of charge | Before we had to photocopy the pregnant woman and maternal and child care card. And mothers had to contribute 100kz or 50kz (0.1-0.2 USD). But this is no longer the case since the program (MCH-HB program) was implemented. (A2) | 10.2% | Intervention characteristics/ relative advantage, outer setting/ patient needs and resources |
| Code | Low complexity for users to aid their understanding | And it's (the MCH-HB is) accessible to any level of education of the mothers, because they have explanations and drawings as well. (A8) | 33.0% |  |
| Upper Category | The appropriate MCH-HB management and supervision at HFs |  | 100.0% |  |
| Lower Category | Adequate training on the MCH-HB for HWs |  | 77.3% |  |
| Code | Adequate ToT | In the seminar that we had there we were only two people, the chief selected herself and one other colleague, so we went there and learned everything about the MCH-HB. (E30) | 19.3% | Inner setting/ readiness for implementation (available resources) |
| Code | Continuous intra-facility training | The refresher is very important and is also welcome, ... those who participate in the seminar are not there permanently, there are several technicians' difficulties. (B21) | 14.8% |  |
| Code | Adequate intra-facility training at the initial phase | To be honest, we didn't encounter any difficulties, because when the booklets came, they taught us properly, they taught us very well. (D42) | 60.2% |  |
| Code | Provision of intra-facility training to all HWs | Yes, before the implementation ...we had time to prepare and better train all the attendants to receive the maternal and child health booklets in order to do a good job. (A1) | 22.7% |  |
| Lower Category | Effective municipality supervision on the MCH-HB in HFs |  | 65.9% |  |
| Code | Motivational supervision for HWs | Supervision motivates me more to work more well. (C21) | 56.8% | Process/ planning, engaging, executing, reflecting and evaluating, characteristics of individuals/ other personal attributes |
| Code | Supervision strengthening HWs' knowledge and skills | And the supervisor evaluates, according to the work he has done, where it is wrong the supervisor corrects, so the technician grows. (E25) | 26.1% |  |
| Lower Category | HWs’ high competency |  | 90.9% |  |
| Code | HWs being competent in the use of the MCH-HB | I think I am confident (in using the MCH-HB), with the seminars that I had I was able to learn a lot and I think I am very confident. (A12) | 80.7% | Characteristics of individuals/ self-efficacy, individual stage of change, other personal attributes |
| Code | HWs being capable of educating users about the MCH-HB | Always when we give the notebook to a mother, whether she is literate or not, we talk to her, we give her a summary of the benefits that the notebook brings, we tell her what she is going to have to do along this journey of the 9 months of pregnancy and the 5 years after birth when the baby is born. (B7) | 64.8% | Characteristics of individuals/ self-efficacy, individual stage of change, other personal attributes |
| Code | HWs having basic knowledge and skills for implementing MCH-HB and related MNCH services | Yes, if by chance the HIV test is positive, we have the drugs that the mother has to take, the HIV positive mother, the effect of two hours in this case, and at the time of the baby's expulsion. (B13) | 25.0% | Characteristics of individuals/ other personal attributes |
| Lower Category | Leadership in HFs |  | 34.1% |  |
| Code | HWs recognizing the responsible person for the MCH-HB | Nurse A is the one responsible for the distribution of the notebook. (E47) | 34.1% | Process/ formally appointed internal implementation leaders |
| Lower Category | Appropriate stock management of the equipment related to the MCH-HB |  | 67.0% |  |
| Code | HFs being provided with adequate equipment to provide the MCH-HB services | Regarding the filling in of the MCH-HB, necessary material was not lacking…. (E4) | 18.2% | Inner setting/ readiness for implementation (available resources) |
| Code | Appropriate MCH-HB stock management | None (the stock management was not difficult), because here we practically already have some stock control sheets where we can put the outputs and inputs. (C2) | 59.1% | Process/ planning, executing |
| Lower Category | Existence of evaluation and the feedback system in HFs |  | 26.1% |  |
| Code | Existence of evaluation and feedback | At the end of the month we do the report. It's the report that's going to tell us, and our graph is in the spreadsheet is what's going to tell us in that month, if our goal was met or we failed. (A17) | 26.1% | Inner setting/ implementation climate (goals and feedback) |
| Upper Category | HFs’ and HWs' positive attitudes toward work |  | 90.9% |  |
| Lower Category | HWs' acceptance to the MCH-HB |  | 85.2% |  |
| Code | Exposure to champions | (Do you know anybody who worked enthusiastically for the MCH-HB?) Yes. He is a dedicated professional, caring and in the right and correct direction of his activities, classified as a best health technician. (D41) | 20.5% | Process/ champions |
| Code | Head of the service being positive to the MCH-HB | Our manager has been a person who has also created enthusiasm in wanting to learn more about filling out the maternal and child health notebook. (E53) | 14.8% | Process/ opinion leaders |
| Code | HWs' positive attitudes toward the MCH-HB | Well, I believe that because of some seminars and also because of what I could see in the notebook itself, it led me to think this way that it is very important, yes. (C15) | 72.7% | Characteristics of individuals/ knowledge and beliefs about the intervention, individual stage of change |
| Code | MCH-HB’s low complexity | With regard to the distribution of the maternal and child notebook, from the boss and the colleagues I did not have any difficulties, everything was in the normality. (E43) | 48.9% | Intervention characteristics/ complexity |
| Lower Category | HWs' positive learning and working attitudes |  | 73.9% |  |
| Code | Attitudes to improve their services | There is always a tendency to want to be good at everything. (D7) | 21.6% | Inner setting/ culture |
| Code | Willingness to exchange ideas | I think it is better when there is this exchange of experience, sometimes you think you do it better than the other and sometimes you don't, so when there is this exchange of experience it is welcome. (C13) | 28.4% | Inner setting/ implementation climate (learning climate) |
| Code | Humble teaching attitudes | Yes, there are methods for that, call the colleague, talk to her humbly, and make her aware of everything and show her the correct point. (A14) | 23.9% | Inner setting/ implementation climate (learning climate) |
| Code | Willingness to seek help and teach each other | I can seek help from a colleague or from my supervisor telling him about my difficulty there, the colleague will help me and I will thank him for what he told me. (C1) | 61.4% | Inner setting/ implementation climate (learning climate) |
| Code | Willingness to serve mothers and children | The goal of my work is to accompany the baby's growth in the best way possible, the more the baby grows the better it is for me. (E40) | 20.5% | Inner setting/ culture |
| Upper Category | User's acceptance and community involvement |  | 73.9% |  |
| Lower Category | Community involvement for the MCH-HB program |  | 15.9% |  |
| Code | Delivery of community education | Before entering the implementation, we did lectures in the churches, to clarify the people in the churches, to announce that there will be a new implementation of the maternal and child health notebook that controls the mother and the baby when it is born. (A16) | 6.8% | Outer setting/ patient needs and resources |
| Code | Local stakeholders’ involvement | The soba (the village leader) should be included in this very work because if in his village he sees a mother and child who is with the MCH-HB and discard it, he could give advice that how they should treat and that the MCH-HB is very important. (C11) | 10.2% | Process/ engaging |
| Lower Category | User's acceptance and adherence to the MCH-HB |  | 70.5% |  |
| Code | Users' acceptance of the MCH-HB | They liked it a lot, they never saw these cards, they liked it so much it's worth it. (E10) | 35.2% | Intervention characteristics/ relative advantage, Outer setting/ patient needs and resources |
| Code | Users' adherence to the MCH-HB | since we started with the maternal and child health booklets there is more of a change, there is more of a drive. Women are now finding out that there is a program after all and they are joining. (B31) | 59.1% |  |

*Parentheses placed after a quotation indicates the interview from which the quotation was derived. A, B, C, D, E indicate five municipalities each.

CFIR: Consolidated Framework for Implementation Research; MCHH: Maternal and Child Health Handbook; HW: Health Worker; HF: Health Facility; ToT: Training of Trainers; MNCH: Maternal, Neonatal and Child Health; HIV: Human Immunodeficiency Virus; USD: United States Dollar; kz: Kwanza (currency of Angola).
