# Supplementary material for: The RE-AIM framework-based evaluation of the implementation of the Maternal and Child Health Handbook program in Angola: a mixed methods study
Source: BMC Health Serv Res. 2022 Aug 22;22:1071. doi: 10.1186/s12913-022-08454-9 (PMC9395902; doi:10.1186/s12913-022-08454-9)
Supplement: Supplementary file 6 — Additional file 6: Supplementary Table 3. Facility-level implementation indicators by location and facility type. [file 12913_2022_8454_MOESM6_ESM.docx]

**Supplementary table. Facility-level implementation indicators by location and facility type**

| Indicator | Definition | Target | All (n=89) | Location | | Facility type | | |
| --- | --- | --- | --- | --- | --- | --- | --- | --- |
|  |  |  |  | Urban area (n=19) | Rural area (n=70) | Hospital (n=6) | Health center (n=28) | Health post (n=55) |
| Reach | | | | | | | | |
| MCH-HB coverage | % MCH-HB distribution among new visitors to antenatal/ delivery/ postnatal care services | 95% | 82.6% | 88.2% | 81.2% | 100.0% | 77.8% | 83.6% |
| Adoption | | | | | | | | |
| Training | Participation in the training of trainers | Yes | 94.4% | 94.7% | 94.3% | 100.0% | 96.4% | 92.7% |
|  | Holding an intra-facility training | Yes | 88.6% | 89.5% | 88.4% | 83.3% | 92.6% | 87.3% |
| Inventory management | Use of inventory management logbook | Yes | 85.2% | 84.2% | 85.5% | 83.3% | 88.9% | 83.6% |
| Mothers' class | Holding mothers' classes every week | Yes | 60.2% | 68.4% | 58.0% | 83.3% | 51.9% | 61.8% |
| Implementation | | | | | | | | |
| MCH-HB retention | % MCH-HB holders at the end of trial among MCH-HB receivers | 90% | 48.8% | 47.1% | 49.3% | 20.0% | 56.0% | 48.1% |
| MCH-HB utilization | % Appropriate birth weight description among MCH-HB receivers | 80% | 32.5% | 47.1% | 28.6% | 40.0% | 40.0% | 28.0% |
| Inventory management | Stock-out | No | 87.5% | 84.2% | 88.4% | 83.3% | 92.6% | 85.5% |
| Mothers' class | Holding mothers' class according to the instruction on themes | Yes | 51.1% | 63.2% | 47.8% | 83.3% | 48.1% | 49.1% |
| Maintenance | | | | | | | | |
| Intra-facility training | Definite person in charge of intra-facility training after the trial | Yes | 76.1% | 84.2% | 73.9% | 83.3% | 85.2% | 70.9% |
| Skills and knowledge | A score of a responsible staff member above the required level | 70/100 | 70.9% | 78.9% | 68.7% | 66.7% | 66.7% | 73.6% |
|  | A median score of staff members above the required level | 60/100 | 80.6% | 100.0% | 73.9% | 100.0% | 73.7% | 81.6% |
| Subjective burden | Subjective burden of a responsible staff member being "low" or "very low" | Yes | 2.3% | 0.0% | 3.0% | 0.0% | 3.7% | 1.9% |
|  | % Subjective burden of staff members being "low" or "very low" | 50% | 8.1% | 12.5% | 6.5% | 0.0% | 15.8% | 5.3% |

*Missing values were excluded.
